# A Penile Spine/Vibrissa Enhancer Sequence Is Missing in Modern and Extinct Humans but Is Retained in Multiple Primates with Penile Spines and Sensory Vibrissae

**DOI:** 10.1371/journal.pone.0084258

**Published:** 2013-12-19

**Authors:** Philip L. Reno, Cory Y. McLean, Jasmine E. Hines, Terence D. Capellini, Gill Bejerano, David M. Kingsley

**Affiliations:** 1 Department of Anthropology, The Pennsylvania State University, University Park, Pennsylvania, United States of America; 2 Department of Computer Science, Stanford University, Stanford, California, United States of America; 3 Department of Developmental Biology, Stanford University School of Medicine, Stanford, California, United States of America; 4 Howard Hughes Medical Institute, Stanford, California, United States of America; University of Florence, Italy

## Abstract

Previous studies show that humans have a large genomic deletion downstream of the *Androgen Receptor* gene that eliminates an ancestral mammalian regulatory enhancer that drives expression in developing penile spines and sensory vibrissae. Here we use a combination of large-scale sequence analysis and PCR amplification to demonstrate that the penile spine/vibrissa enhancer is missing in all humans surveyed and in the Neandertal and Denisovan genomes, but is present in DNA samples of chimpanzees and bonobos, as well as in multiple other great apes and primates that maintain some form of penile integumentary appendage and facial vibrissae. These results further strengthen the association between the presence of the penile spine/vibrissa enhancer and the presence of penile spines and macro- or micro- vibrissae in non-human primates as well as show that loss of the enhancer is both a distinctive and characteristic feature of the human lineage.

## Introduction

 The rapid accumulation of sequence information from many species is making it possible to search for genomic events associated with lineage-specific phenotypes [1,2]. We previously reported that humans have an approximately 60 kilobase (kb) genomic deletion located downstream of the Androgen Receptor (AR) gene on the X chromosome [3]. This deletion (hCONDEL.569) removes an otherwise highly conserved mammalian enhancer located 218 kb and 300 kb from the *AR* transcriptional start sites of chimpanzees and mice, respectively. The orthologous enhancer from both species consistently drives gene expression in penile spines and vibrissae in transgenic mice. Androgen signaling is known to play a key role in development of both structures as castration or mutational inactivation of the *AR* gene results in and the loss of penile spines in rodents and primates and reduced growth of vibrissae in male mice [4-6]. Interestingly, both penile spines and vibrissae are structures that, while present in chimpanzees, macaques, and mice, are missing in humans. Thus, there exists strong correlative evidence that loss of these structures is related to this genomic deletion that occurred in the human lineage since our divergence from chimpanzees.

 Although humans are usually described as lacking penile spines [7], a significant fraction of human males develop small white papules on the penis, variably known as Tyson's glands, papillae of the penis, acral angiofibromas, and pearly penile papules (PPP) [8-10]. Some researchers have speculated that PPP could be evolutionary vestiges of penile spines [11]. In addition, although penile spines have also been reported both morphologically and histologically in chimpanzees [12], the structures are rarely studied and could conceivably be polymorphic in chimpanzees or other ape lineages. To further examine whether loss of the penile spine/vibrissa enhancer is polymorphic in humans and other primates, we survey for the relevant enhancer sequences in large numbers of human individuals, the recently available Denisovan genome [13,14], a panel of chimpanzee and bonobo individuals, and in the genomes of gorillas, orangutans, gibbons, rhesus, marmoset, and bushbaby. We find that the enhancer sequence is missing from all modern and extinct human genomes tested, but is present in all non-human primates examined, confirming that the enhancer deletion arose early in the human lineage and is not deleted in multiple primates that still retain penile spines and vibrissae. 

## Materials and Methods

### Ethics Statement

 Human DNA samples used in this study were either purchased from Clontech (#636401) or collected by Dr. Mark Shriver [Pennsylvania State University (PSU); with approval from the PSU Institutional Review Board]. Non-human primate DNA was either purchased from the Coriell Cell Repositories (Camden, NJ) or came from previously existing samples purified from surplus clinical specimens collected during routine veterinary care. The PSU Office of Research Protections has concluded the use of these samples is exempt from IACUC review and consistent with the objective of minimizing the number of non-human primates used for research endeavors. 

### Trace Archive Analysis

 All human traces in the NCBI Trace Archives as of February 2011 were tested for their ability to align to the 4,839 base pair (bp) chimpanzee enhancer (panTro3.chrX:67,539,730-67,544,568) using LASTZ [15] with the parameters T=0 W=6 Q=HoxD55.q K=1800 M=0 L=5400. A hit was defined as any read mapping to the non-repetitive regions of high conservation at chimpanzee coordinates panTro3.chrX:67,541,951-67,543,228. No reads satisfied these mapping criteria.

 All gorilla whole genome trace sequences were queried with the 4,839 bp chimpanzee enhancer. The ten highest-scoring traces tiled, but the eleventh highest-scoring trace was separated from the others by a partially repetitive region of no detected homology. To determine the gorilla DNA sequence separating these traces, 1.25 kb of gorilla and chimpanzee sequence (corresponding to panTro3.chrX:67,542,933-67,544,183) was amplified with Phusion polymerase using primers 5’-GGGGGAAGCAGCACACAGTGG-3’ and 5’-ACCACGCCTGGCCTGATCCT-3’ and PCR parameters of 35 cycles, 63 C annealing temperature, and 2.5 min extension time. The resulting sequence was inserted into Zero Blunt TOPO PCR Cloning vectors, and one chimpanzee and three gorilla clones were sequenced using primers 5’-GTAAAACGACGGCCAG-3’ (M13F-20) and 5’-CAGGAAACAGCTATGAC-3’ (M13R). In each case we confirmed the sequences belonged to the appropriate species by verifying predicted base pair differences from the chimpanzee reference genome and gorilla traces. The eleven traces and generated gorilla sequence data were merged into a single contig with discrepant base calls resolved by majority vote with ties broken by minimizing differences with the chimpanzee reference genome. The resulting gorilla contig was used as input into the primate multiple alignment. The new gorilla sequences have been deposited in GenBank (accession numbers: KF638584-KF638590).

### 1000 Genomes Analysis

 All Illumina and 454 nucleotide sequence reads available from the November 2010 release of the 1000 Genomes Project data were aligned to the full hCONDEL.569 chimpanzee DNA construct using BWA [16] with its default parameters. Reads were examined for hits in the non-repetitive regions of high conservation at chimpanzee coordinates panTro3.chrX:67,541,951-67,543,228 after filtering for non-gapped alignments at least 35 bp in length with, at most, one mismatch. Paired-end reads were restricted to have a properly oriented mate pair mapping. No reads satisfied these mapping criteria.

### Multiple Alignment Build

A chimpanzee-centric multiple alignment was generated with the following species (genome assemblies): chimpanzee (panTro3), bonobo (panPan1), gorilla (the constructed contig described above), orangutan (ponAbe2), gibbon (nomLeu3), rhesus macaque (rheMac3), marmoset (calJac3), bushbaby (otoGar3), and mouse (mm10). Chimpanzee and mouse sequences used as input to the alignment were the enhancer regions tested previously [3]: panTro3.chrX:67,539,730-67,544,568 and mm10.chrX:98,450,137-98,457,790. Input sequences for the other non-gorilla species were identified by using BLAT [17] on the genome sequences with the chimpanzee sequence as the query and padding the highest-scoring region by 100 bp on each flank.

 Pairwise alignments between chimpanzee and all other species were generated using LASTZ with parameters T=0 W=6 and passing the output through the lav2maf and single_cov2 programs. The chimpanzee-centric multiple alignment was generated using roast [15] with default parameters. 

### PCR Analysis

DNA samples for 25 chimpanzees (*Pan troglodytes*) and 3 bonobos (*Pan paniscus*) were obtained from the Coriell Cell Repository (Table S1). An additional 19 chimpanzee [18] and a single gorilla (*Gorilla gorilla*) samples were provided as a gift from Dr. George Perry (PSU). Dr. Anne Stone (Arizona State University) kindly provided 2 additional gorilla DNA samples. The 2 orangutan (*Pongo pygmaeus*) and single gibbon (*Hylobates agilis*) samples were gifts from Dr. Shriver. Each PCR was performed with Phusion polymerase containing 10 ng of chimpanzee, bonobo, human, 1:1 chimpanzee-human mixed DNA, or a water control. The ancestral chimpanzee sequence was targeted using primers 5’-TATGCCACTTTCTTCATGTTGTGGG-3’ and 5’-AAAGCCATAAGTGCCTAGGTGTAGG-3’, and the derived human allele was amplified using primers 5’-ATGCGGTTGGCAATAGCTAAACTAG-3’ and 5’-AGCAAAGGCAGGAAGACTCCAAGTG-3’. An equal mix of the ancestral and derived primer pairs was included in each reaction using PCR parameters of 35 cycles, 63 C annealing temperature, and 2 minute extension time. 

## Results

### No evidence of the AR enhancer in the human population

**Figure 1:**
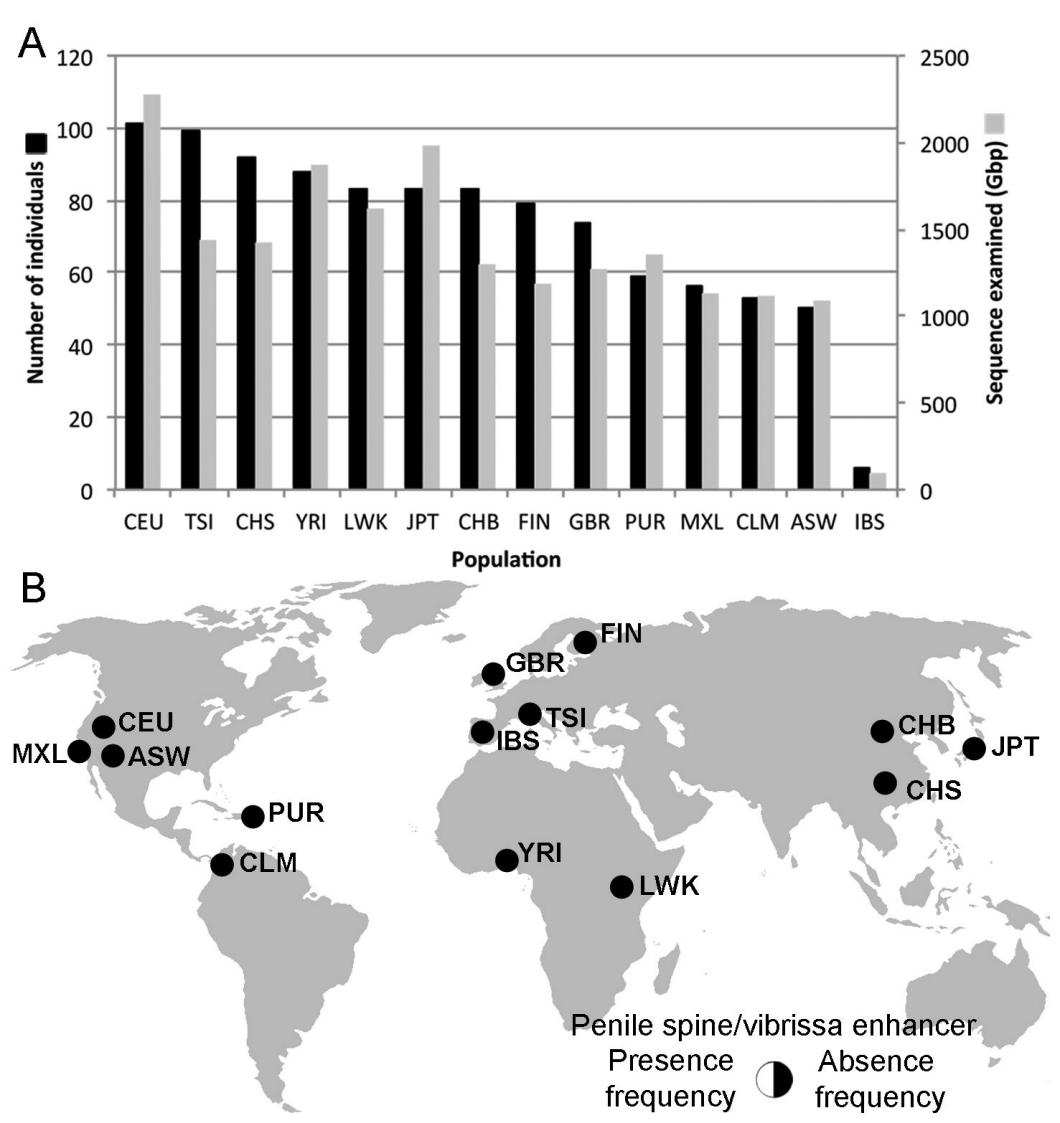
Survey of human populations for presence or absence of the *AR* penile spine/vibrissa enhancer sequences within hCONDEL.569. A) Number of individuals surveyed (black bars) and total billions of base pairs (Gbp) examined (gray bars) from 14 different human populations. B) No sequences matching the chimpanzee enhancer were found in the worldwide sample of 1,006 individuals, indicating fixed deletion of the AR penile spine/vibrissa enhancer in modern humans. The populations from the Americas are substantially displaced and/or admixed. CEU, Utah residents with ancestry from northern and western Europe; TSI, Toscans in Italy; CHS, Han Chinese South; YRI, Yoruba in Ibadan, Nigeria; LWK, Luhya in Webuye, Kenya; JPT, Japanese in Tokyo, Japan; CHB, Han Chinese in Beijing, China; FIN, Finnish in Finland; GBR, British from England and Scotland; PUR, Puerto Ricans in Puerto Rico; MXL, Mexican Ancestry in Los Angeles, CA, USA; CLM, Colombians in Medellín, Colombia; ASW, African Ancestry in SW USA; IBS, Iberian Populations in Spain.

 Our current search of the NCBI Trace Archives contained 360,955,139 traces spanning 206,599,378,641 bp, representing an increase of more than 146,000,000 traces over previous surveys [3]. We found no evidence for the ancestral sequence in any human sequence in the repository. To supplement these data with human sequence that has associated population information, we searched the available 1000 Genomes data comprising 19.2 terabases of sequence from 1,006 people from 14 populations for evidence of the ancestral conserved sequence hCONDEL.569 (Figure 1A). This included sequence from 8 individuals previously assayed via PCR [3]. We found no evidence of the ancestral allele in any 1000 Genomes data analyzed (Figure 1B). We also investigated genome alignments between chimpanzee and short-read sequences extracted from extinct hominids, Neandertals [19] and Denisovans [14], and found no evidence of the ancestral allele in either extinct hominid population. All analyses performed thus far indicate fixation of the hCONDEL.569 deletion within all humans.

### Survey of the AR enhancer sequence in chimpanzees and bonobos

**Figure 2:**
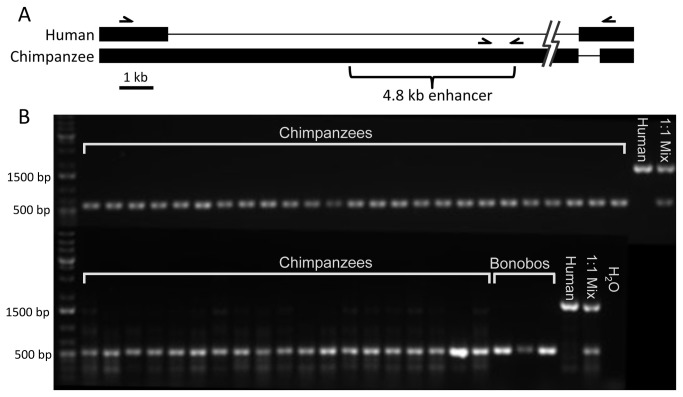
PCR screen of chimpanzees and bonobos. A) Alignment of human and chimpanzee genomic sequences. Thick bars indicate existing sequences; narrow lines indicate sequence gaps due to deletions or insertions. Half-arrows indicate the primer pairs used to assay the region. The bracket identifies the chimpanzee DNA previously tested for enhancer activity. The jagged lines indicate a 50 kb break to maintain scale. The primers targeting the chimpanzee enhancer sequence amplify 535 bp that are not expected to be present in the human genome. The primers spanning the deletion breakpoints will amplify 1,674 bp of human sequence, but are separated by over 60 kb in the chimpanzee. B) PCR results of the chimpanzee and bonobo DNA samples. All cases confirm the presence of the conserved enhancer with no evidence of a human-like deletion.

 The *AR* enhancer is present in the reference genome assemblies of the chimpanzee and bonobo. To test for possible polymorphism of an enhancer deletion in the *Pan* lineage, we conducted a PCR screen of chimpanzee and bonobo DNA samples. The chimpanzee samples comprised both wild (n=20) and captive-born (n=24) individuals (Table S1). The bonobos (n=3) were captive-born individuals from three separate families. To simultaneously test for the presence of the enhancer and deletion, we performed a double PCR assay combining separate primer pairs targeting the chimpanzee and human alleles (Figure 2A). One pair targeted 535 bp within the 4.8 kb chimpanzee sequence previously shown to display enhancer activity. The other pair amplified 1,674 bp of human sequence spanning the deletion break point using primers designed to identical human and chimpanzee reference sequences. This set also spanned a 622 bp human-specific insertion at the break point. Neither primer pair is expected to amplify sequences from the alternative genotype due to either loss of the sequence in the derived human allele or the greater than 60 kb distance between the shared primers in the chimpanzee. The ability of these primer pairs to operate simultaneously was confirmed in reactions containing a 1:1 mix of human and chimpanzee genomic DNA.

 The presence of the enhancer sequence was confirmed in all cases for both species of *Pan* (Figure 2B). Furthermore, no copies of the derived human-size allele were observed in any cases. These results establish that the *AR* enhancer sequence occurs at high frequency and its presence is likely fixed in both chimpanzee and bonobo populations. 

### Confirmation of the ancestral sequence in other species of apes

**Figure 3:**
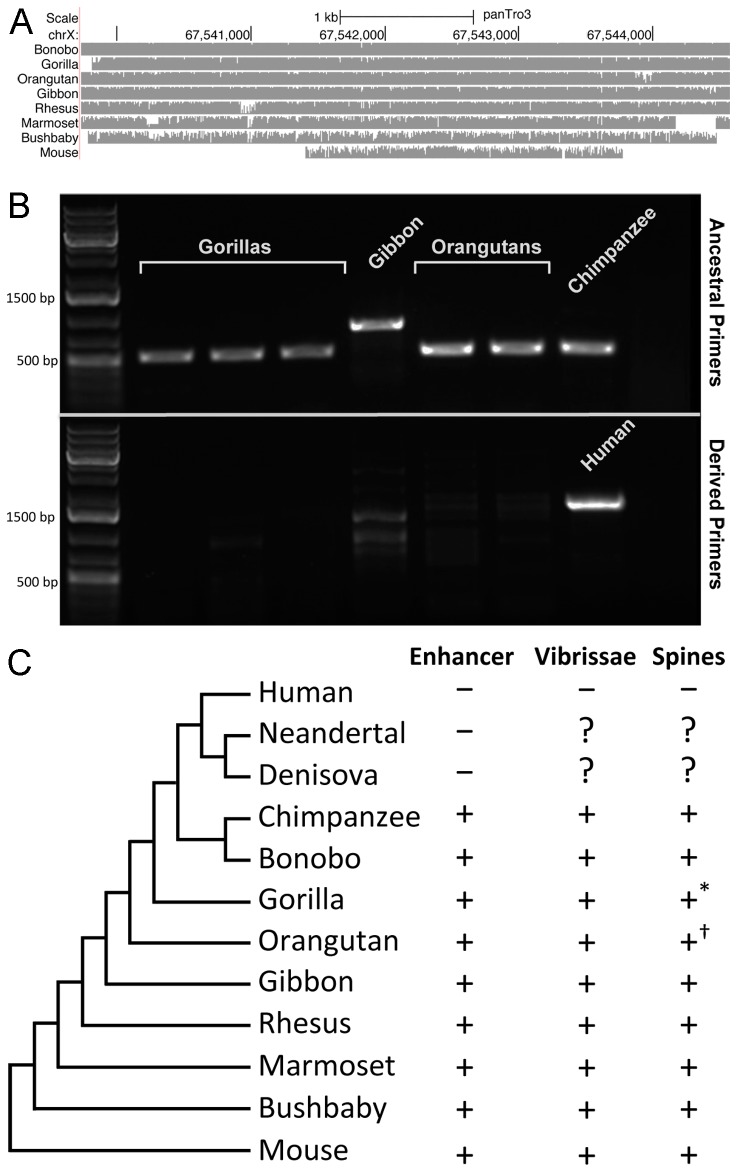
Presence of the *AR* enhancer in non-human primates. A) Multiple alignment of the 4,839 bp enhancer in extant non-human primates and mouse. B) PCR confirmation of the presence of the ancestral *AR* enhancer in gorillas, a gibbon, and orangutans. The ancestral primer pair amplifies predicted products (including a ~450 bp AluY and assembly gap insertion in gibbon) in each of the ape species. The derived (human) primers fail to amplify a corresponding product in any of the apes. C) Phylogeny of the species examined in this study and status of *AR* enhancer and penile spines. ? Presence/absence of penile spines is not known for the extinct hominids, * Juvenile gorillas likely have penile spines [23,28], † Orangutan specimens have ‘hinged platelets’ that may [22] or may not [23] be homologous to penile spines.

 To further characterize the distribution of this *AR* enhancer in additional primates, we analyzed the reference genomes of the gorilla, orangutan, gibbon, rhesus, marmoset, and bushbaby. Maintenance of the conserved sequence is evident in all reference genomes except for gorilla. Given the conservation profile of the enhancer within the primate lineage, it seemed possible that the absence of the enhancer in the reference gorilla genome was an artifact due to the use of human homology as input to the gorilla genome assembly pipeline [20]. To investigate this possibility, we examined gorilla whole genome shotgun reads in the Trace Archives. The Trace Archive contains clear evidence of gorilla DNA reads that show homology to the conserved *AR* enhancer (Figure S1). We assembled a gorilla sequence spanning the entire enhancer using a combination of trace reads and experimental amplification and sequencing from gorilla DNA (see Methods). A multiple alignment of this region with the reference genome assemblies of the other species demonstrates the strong conservation of the enhancer within non-human primates (Figure 3A).

To further confirm the presence of the *AR* enhancer in the extant hominoids, we performed PCR on DNA from 3 gorillas, 2 orangutans, and 1 gibbon using the ancestral and derived primer pairs described above. We confirmed the presence of the enhancer sequence in all non-human primate samples (Figure 3B). In the two great apes, the PCR product was similar in length to those of the chimpanzee and bonobo (535 bp) and matched the predicted lengths from the reference genome or constructed contig. In the case of the gibbon, we amplified a substantially longer product, in agreement with the presence of ~450 bp of inserted sequence (including a 301 bp AluY and a 100 bp gap between assembly scaffolds) in the gibbon reference genome that results in an approximately 1000 bp product.

## Discussion

 Our previous analysis suggested a strong association between the evolutionary loss of an *AR* enhancer and the phenotypic loss of penile spines and vibrissae in humans. This association could be weakened if 1) the enhancer and/or penile spines still occurred at a significant frequency in modern humans or 2) the enhancer, penile spines, and vibrissae were not generally common in non-human primates. The current analysis provides compelling evidence that the deletion is virtually fixed in humans. Given the absence of corresponding sequences from Neandertals and Denisovans, the characteristic human deletion likely arose more than 800,000 years ago and potentially prior to the origin of the *Homo* lineage from ancestral hominids. While the penile spine/vibrissa enhancer is deleted in all humans examined, various reports indicate 15-50% of human males develop small white growths on the penis [9,21]. Though the causes of these benign growths are still unknown, their histological structure is distinct from the innervated keratinized spines found in non-human primates. PPP most closely resemble acral angiofibromas that can form at other sites in the body [8], and their variable presence as a common anatomic variant in males is clearly unrelated to potential human polymorphism for the penile spine/vibrissa enhancer. 

In contrast to humans, we find that the penile spine/vibrissa enhancer is present in all chimpanzees and bonobos tested, as well as in the gorilla, orangutan, gibbon, rhesus, marmoset, and bushbaby genomes. Each of these species also possesses sensory vibrissae and keratinized, innervated penile spines or platelets at least at some point during their life history (Figure 3C) [5,12,22-27]. These data strengthen the correlation between the presence of the enhancer and the presence of penile spines and sensory macro- or micro- vibrissae in most primates. Functional experiments are still required to test whether deletion of this single enhancer can disrupt the formation of penile spines and vibrissae, a molecular event that may help explain the phenotypic loss of these structures in the human lineage. 

## Supporting Information

Table S1 **Specimen numbers and sources of chimpanzee (**Pan** troglodytes) and bonobo (**Pan** paniscus) specimens used in the PCR.**
(XLSX)

Figure S1 **Gorilla contig assembled across the *AR* enhancer region.** Chimpanzee assembly panTro3 browser shot showing the location of the enhancer sequences used to search the gorilla Trace Archive data (blue bar), raw gorilla trace data, additional sequences experimentally amplified from gorilla DNA, and the resulting gorilla enhancer contig constructed from the trace and experimental sequence reads. Black indicates base pairs identical to chimpanzee, red lines indicate single nucleotide differences, orange lines indicate gorilla nucleotide insertions, hatched double lines indicate gaps. Assembled gorilla contig available as GenBank KF638584.
(TIF)
