# Supplementary figures and images for: A Penile Spine/Vibrissa Enhancer Sequence Is Missing in Modern and Extinct Humans but Is Retained in Multiple Primates with Penile Spines and Sensory Vibrissae
Source: PLoS One. 2013 Dec 19;8(12):e84258. doi: 10.1371/journal.pone.0084258 (PMC3868586; doi:10.1371/journal.pone.0084258)

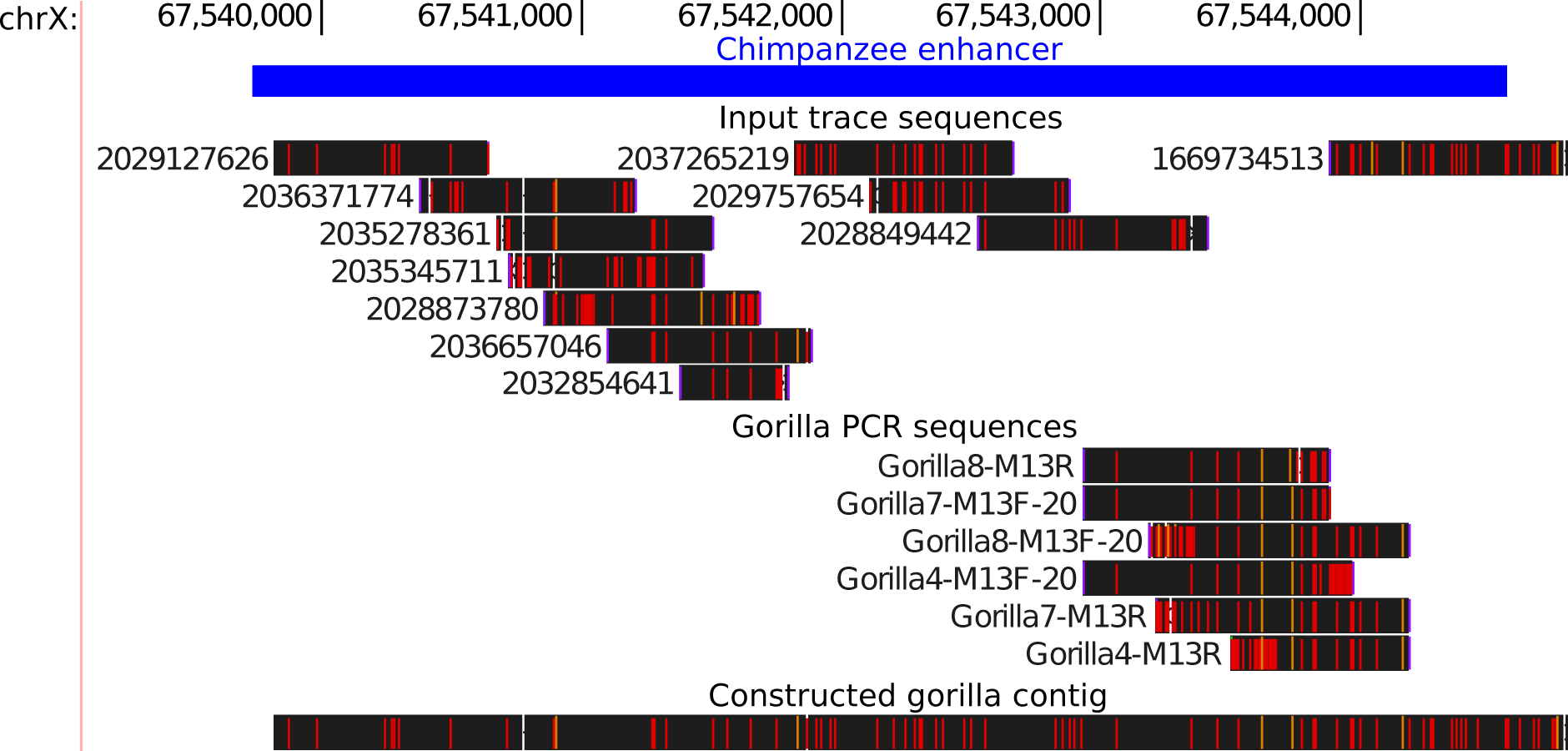

Supplement: Figure S1 — Gorilla contig assembled across the AR enhancer region. Chimpanzee assembly panTro3 browser shot showing the location of the enhancer sequences used to search the gorilla Trace Archive data (blue bar), raw gorilla trace data, additional sequences experimentally amplified from gorilla DNA, and the resulting gorilla enhancer contig constructed from the trace and experimental sequence reads. Black indicates base pairs identical to chimpanzee, red lines indicate single nucleotide differences, orange lines indicate gorilla nucleotide insertions, hatched double lines indicate gaps. Assembled gorilla contig available as GenBank KF638584. (TIF) [file pone.0084258.s002.tif]
